# Predictive value of tissue p53 protein expression and serum p53 antibodies in oral potentially malignant disorders: Relative to oral squamous cell carcinoma

**DOI:** 10.1016/j.jtumed.2021.11.008

**Published:** 2021-12-31

**Authors:** Abbas S. Khan, Sajjad Ahmad, Zahoor Ullah, Naveed Sadiq, Mohsina Haq, Ahmareen K. Sheikh

**Affiliations:** a Department of Oral Pathology, Peshawar Dental College, Warsak Road, Peshawar, Khyber Pakhtunkhwa, Pakistan; b Department of Pathology, Peshawar Medical College, Riphah International University, Islamabad, Pakistan; c Department of Biochemistry, Peshawar Medical College, Riphah International University, Islamabad, Pakistan; d Institute of Public Health & Social Sciences, Khyber Medical University, Peshawar, Pakistan; e Department of Microbiology, Peshawar Medical College, Riphah International University, Islamabad, Pakistan; f Department of Pathology, Pakistan Institute of Medical Sciences, Islamabad, Pakistan

**Keywords:** اضطرابات الفم الخبيثة المحتملة, سرطان الخلايا الحرشفية الفموي, بروتين مثبط الورم 53, الكيمياء الهيستولوجية المناعية, مقايسة الممتز المناعي المرتبط بالإنزيم, الأجسام المضادة, القيمة التنبؤية للاختبارات, Antibodies, Immunohistochemistry, Oral potentially malignant disorders, Oral squamous cell carcinoma, Predictive value of tests, Tumour suppressor protein p53

## Abstract

**Objective:**

This study aims to assess and report the predictive value of tissue p53 protein expression and serum p53 antibodies as a screening tool for oral potentially malignant disorders (OPMDs) cases with risk of malignant transformation.

**Methods:**

A case–control study was jointly conducted at the Department of Pathology and Oral and Maxillofacial Surgery in several dental institutes in the country from April 2016 to March 2017. A total of 180 eligible subjects (60 cases of OPMDs, 60 cases of oral squamous cell carcinoma, and 60 controls) were included in the study. Tissue p53 immunoreactivity was determined by immunohistochemistry, and serum concentrations of p53 antibodies were determined by enzyme-linked immunosorbent assay. Specimens were collected for laboratory investigations after obtaining written consent from both patients and controls.

**Results:**

Among the study participants, the recorded male to female ratio was close to 2:1, and most participants fell in the age range of 41–60 years and above. Of the 60 cases of OPMDs, the observed tissue p53 immunopositivity was 73.3% (n = 44) while for the p53 antibody, the seropositivity was 96.7% (n = 58). The sensitivity for p53 immunoreactivity was 73%, and specificity was 98.3% between OPMDs and healthy individuals.

**Conclusion:**

The present study provides evidence (for OPMDs) that serum p53 antibodies and p53 immunoreactivity could be used as a sensitivity test and a specific test, respectively, and may contribute to determining the potential of OPMD for malignant transformation risk.

## الملخص

**أهداف البحث:**

لتقييم والإبلاغ عن القيمةالتنبؤية المحتملة لتعبير البروتين 53 (بـ53) فى ا لأنسجة والأجسام المضادة له في الدم كأداة فحص في حالات اضطرابات الفم الخبيثة المحتملة لخطر التحول الخبيث.

**طرق البحث:**

تم إجراء دراسة مراقبة الحالة بشكل مشترك في أقسام علم الأمراض وجراحة الفم والوجه والفكين في معاهد طب الأسنان المختلفة في البلاد من أبريل ٢ ۰ ١٦ إلى مارس ٢ ۰ ١ ۷. تم انضمام مجموعة من ١٨ ۰ شخصا مؤهلا (٦ ۰ حالة من حالات اضطرابات الفم الخبيثة المحت ملة وسرطان الخلايا الحرشفية الفموي لكل منهما و ٦ ۰ حالة تحكم ) لأخذ عينات الأنسجة والمصل. تم تحديد نشاط المناعة لبروتين 53 في الأنسجة عن طريق الكيمياء الهيستولوجية المناعية وتم تحديد تركيزات الأجسام المضادة لبروتين 53 في المصل عن طريق مقايسة الممتز المناعي المرتبط بالإنزيم. تم جمع العينات للقياسات المعملية بعد الحصول على موافقة خطية من كل من الحالات والضوابط.

**النتائج:**

من بين المشاركين في الدراسة، كانت نسبة الذكور إلى الإناث المسجلة قريبة من ٢: ١ وفي الغالب في الفئة العمرية من ٤١ إلى ٦ ۰ عاما وما فوق. من بين ٦ ۰ حالة من حالات اضطرابات الفم الخبيثة المحت ملة، كانت الإيجابية المناعية لبروتين 53 في الأنسجة ۷ ٣.٣ ٪ ( عدد =٤٤/٦ ۰ ) وبالنسبة لل أجسام المضاد ة، كانت الإيجابية المصلية ۹ ٦. ۷ ٪ (عدد =٥٨/٦ ۰). بين حالات اضطرابات الفم الخبيثة المحت ملة والأفراد الأصحاء ، كان اختبار نشاط المناعة لبروتين 53 ذو حساسية ۷ ٣ ٪ و خصوصية ۹ ٨.٣٪.

**الاستنتاجات:**

قدمت الدراسة الحالية دليلا على أنه بالنسبة ل حالات اضطرابات الفم الخبيثة المحت ملة، يمكن استخدام الأجسام المضادة لبروتين 53 في الدم كاختبار حساسية ويمكن استخدام اختبار نشاط المناعة لبروتين 53 كاختبار ذا خصوصية وقد يساهم في تحديد حالات اضطرابات الفم الخبيثة المحت ملة مع احتمال خطر التحول الخبيث.

## Introduction

Oral cancer is a remarkable component of the global cancer burden, with increased morbidity and mortality. Oral squamous cell carcinoma (OSCC) is a common histopathological variant of oral epithelial malignancy.^1^ Oftentimes it is caused by numerous potentially malignant disorders.^2^ The oral potentially malignant disorders are clinically detectable oral mucosal disorders that carry an increased risk of developing oral malignancy. The reported worldwide prevalence of oral potentially malignant disorders is about 4.47%.^3^ The OPMDs are characterized by diverse forms of clinical presentations, which either regress or progress to OSCC. Clinically, they appear as white, red, or mixed (red and white) lesions marked as oral leucoplakia, oral lichen planus, oral erythroplakia, snuff dipper keratosis, oral submucous fibrosis, and others.^2^ Histologically, OPMDs present themselves as epithelial precursor lesions, characterised by squamous cell hyperplasia, with or without other specific cytological and architectural alterations termed as oral epithelial dysplasia (OED), subcategorised as mild, moderate, or severe, dysplasia, and carcinoma in situ.^3^^,^^4^ As noted by observational studies, the risk of malignant transformation among OPMDs varies from lesion to lesion. For example, the reported malignant transformation rate (MTR) is 0.13%–42.2% for oral leukoplakia, up to 70% for oral proliferative verrucous leukoplakia, and 0–10% for oral lichen planus.^5^^,^^6^ Early stage diagnosis of an OPMD is key for preventing malignant transformation in the disease and can therefore decrease the morbidity and mortality of OSCC. To improve the prognosis of OSCC, it is vital to explore a biomarker that can be employed to predict the possible risk of malignant transformation of an OPMD. This goal may be achieved by investigating a biomarker in the tissue or body fluid samples of OPMDs cases with a predicting property of the development of OSCC.^7^, ^8^, ^9^ Inactivation of tumour suppressor genes (TSGs) is one of the reported key episodes in the multistep process of developing oral malignancy and premalignancy.^10^ Among the TSGs associated with oral cancers and oral potentially malignant precursors, p53 is the highly searched gene.^10^^,^^11^ The role of p53 is noteworthy, as, in the research literature, it has been designated as the ‘molecule of the year’,^12^ as an ‘apoptotic super hero’,^13^ ‘the guardian of genome, policeman of oncogenes’,^14^ and as the ‘caretaker and gatekeeper gene’.^15^ The tumour suppressor functions of p53 are achieved via cell cycle arrest, repair of DNA, senescence, and apoptosis.^12^, ^13^, ^14^, ^15^ Researchers have marked the aberrations in the p53 gene, resulting in accumulation of p53 protein in the tissue samples of oral cancerous and precancerous lesions, which in turn leads to induction of p53 autoantibodies in circulation as a part of humoral immune response.^16^, ^17^, ^18^, ^19^

Therefore, following this research, p53 was selected, tissue p53 immunoreactivity and p53 seropositivity were evaluated, and its clinical usefulness as a screening tool for MT in OPMDs cases was analysed, which in turn will improve the OSCC prognosis via early stage diagnosis. The alternate hypothesis of the study was that immunohistochemical expression of the p53 protein and p53 autoantibody levels are different in tissue and serum samples of OPMDs, OSCC, and healthy individuals, and they are effective predictors of the malignant potential of an OPMD.

## Materials and Methods

A case–control study was conducted on 180 subjects, comprising 120 cases (60 cases of OPMDs and OSCC each) and 60 healthy individuals. The data collection involved multi-dental care centres in Pakistan, including the oral and maxillofacial surgical units of the Peshawar Dental College (PDC), the Khyber College of Dentistry (KCD), and the Pakistan Institute of Medical Sciences (PIMS). Laboratory diagnoses were conducted at the Pathology Department of the Peshawar Medical College (PMC) from 3 April 2016 to 31 March 2017. Data from both cases and controls were collected through a non-probability purposive sampling technique from the outpatient departments of the participating centres. The cases were defined as patients with OPMD and OSCC manifestation for which an excision biopsy was warranted in any case. The controls were defined as healthy patients who visited the maxillofacial departments of the participating centres for the surgical extraction of third molar impactions. The rationale behind choosing these controls was to avoid ethical dilemmas for obtaining surgical biopsy in otherwise healthy individuals. Written consent was obtained from all cases and controls in this study. Ethical permission to conduct this study was obtained from the Institutional Review Board of Prime Foundation Pakistan.

A structured pro forma was used as a data collection tool to record the detailed history of the study participants. The study participants were interviewed before the collection of serum and tissue bio-samples. In addition to the criteria of recruiting only histopathologically diagnosed cases of OPMDs and OSCC, the inclusion criteria included patients who had not yet received any treatment for oral malignancy and premalignancy. Individuals who failed to provide informed consent due to lack of interest, as well as subjects with co-existing medical illnesses, such as liver cirrhosis, acute and chronic pancreatitis, and diabetes, were excluded from the study.^20^ The healthy individuals were those who consented to the study participation and visited the recruiting centres for dental treatments of 3rd molar surgical extractions, alveoloplasty, and others, in which an extra portion of normal oral mucosal tissue was removed and intended to be discarded during the procedure.^21^ Beside the tissue samples, 5 mL of venous blood was collected from all the patients under aseptic conditions and stored in a disposable, non-pyrogenic gel tube with clot activating vacutainer (Atlas Medo-O-VAC Fransico). The blood was centrifuged (15 min at 1000×*g*) before serum was collected and placed in an Eppendorf tube to be kept at −70 °C. Serum p53 antibodies were assessed via ELISA by strictly following the manufacturer's instructions for the anti Pp53 ELISA kit (Elabscience, Wuhan; China-Catalog No: E-EL-H0910).^22^ The reported detection range of the ELISA kit was 78.125–5000 pg/mL. The concentration of p53 antibodies was measured in pg/mL using an automated microplate reader (Heales-MB 580, China) set at 450 nm. Serum samples were run in duplicate. The observations were marked as positive for p53 Ab status with values of 401 pg/mL and above, and negative p53 Ab status values ranged from 0 to 400 pg/mL. The cut-off value was estimated via 95% CI of the mean (Mean ± 2SD; lower limit: 310.01, upper limit: 370.68; margin of error: 30.3).^22^ The oral mucosal tissues were processed and stained with haematoxylin and eosin for histopathological slide review. H&E staining confirmed the diagnosis of OPMDs and OSCC in the tissue samples, while p53 staining was evaluated by immunohistochemistry using a semi-quantitative scoring system. Special grip-coated slides (Dako Flex IHC Microscope slides) were used for immunohistochemical staining of the tissue samples of the study participants with p53 protein antibody (Clone: DO-7; Antibody type: Monoclonal mouse, Dako, Denmark). The protocol employed for scoring p53 immunoreactivity included marking the OPMD and OSCC tissue specimen slides either positive or negative. The basic criteria for positive staining were the presence of a clear brown nuclear stain. The percentage of stained nuclei was assessed by enumerating p53 stained cells per 100 tumour, dysplastic, or hyperplastic epithelial cells, in the area of best staining with a cut-off value of 10% nuclei stained with p53 immunohistochemically. The p53 stained nuclei counts were categorised into the following four categories: absence of staining or occasional keratinocyte staining (−), keratinocytes staining of 10–33% (+), keratinocytes staining of 34–66% (++), and keratinocytes staining greater than 66% (+++). The staining intensity was subjectively graded into definite but light stain (1+), darker stain (2+), and most intense staining (3+).^23^^,^^24^ In the tissue sections of normal oral mucosa, p53 stained nuclei counts were categorised into the following two categories: the negative stain comprised the absence of expression of p53 protein detected in any epithelial nuclei or even rare cells positive (1–10 cells per section), while the positive p53 immunohistochemical stain was marked when clear brown coloured staining with more than 5% of suprabasal cells showed positivity.^25^, ^26^, ^27^ In epithelial tissue specimens of OPMDs and healthy individuals, p53 staining confined exclusively to basal layers was considered normal expression and marked as a negative case. Endometrioid carcinoma was used as a positive control for p53 immunoreactivity.

The sample size for this study was estimated using the Stata (Software for Statistics and Data Science, for a power of 80% and 95% confidence interval (CI) for a 2-tail hypothesis with a 1:1 ratio. The data were analysed using SPSS version 20. The percentages were calculated for each categorical variable and the chi-square test was applied for statistical significance, where appropriate. A probability value of less than or equal to 0.05 was considered statistically significant.

## Results

**Figure 1:**
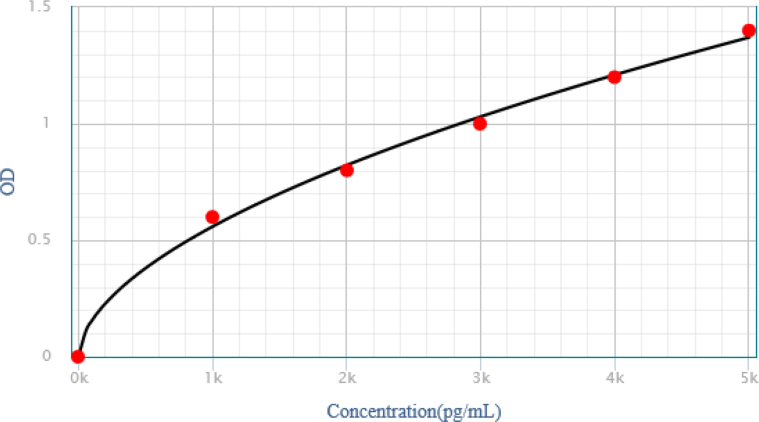
Standard curve: p53 ELISA (OD [450 nm] vs Concentration [ng/mL]).

The results of the present study are summarised in Table 1, Table 2, Table 3, Table 4 shown below, each with an essential description (see Figure 1).

The age of OPMD cases ranged from 30 to 95 (54.5 ± 14.41). Most of the cases of OPMDs (n = 33) and OSCC (n = 35) were older than 50 years. The recorded male to female ratio for OPMDs, OSCC, and healthy individuals was approximately 2:1. The mean serum p53 antibody levels were highest in OSCC cases, followed by OPMDs, and lowest among the controls. Among OPMDs, circulating serum p53 antibodies were detected in 58 out of 60 cases. The serum p53 antibody levels above the cut-off level among healthy individuals were not more than 600 pg/mL, as compared to cases of OPMDs (maximum 3898 pg/mL) and OSCC (maximum 4249 pg/mL). Among cases of OPMDs, tissue p53 immunoreactivity was observed in 44/60 cases while 51 out of 60 cases of OSCC expressed p53 protein expression in the tissue samples of malignant lesions.

Among OPMD cases, 96.7% (n = 58) were p53 Ab seropositive, and among them, 70% (n = 42) expressed p53 protein immunohistochemically in preneoplastic cells. Among healthy individuals, 20 out of 60 subjects expressed p53 Ab in serum, of which only one subject expressed p53 protein in the suprabasal layers of normal oral mucosa (Table 1).

**Table 1:** Description of age, gender, serum p53 antibody concentration, and tissue p53 immunoreactivity of the study subjects.

Study variables	Study groups			*p*-value∗
	OPMD	OSCC	Healthy subjects	
**Age**				
Mean ± SD	54.5 ± 14.4	55.0 ± 14.4	50.0 ± 11.8	0.230^a^
**Age groups, n (%)**				
20–40 years	10 (16.7)	12 (20.0)	14 (23.3)	0.256^b^
41–50 years	17 (28.3)	13 (21.7)	19 (31.7)	
51–60 years	10 (16.7)	16 (26.7)	16 (26.7)	
>60 years	23 (38.3)	19 (31.7)	11 (18.3)	
**Sex, n (%)**				
Male	40 (66.7)	32 (53.3)	32 (53.3)	0.232^b^
Female	20 (33.3)	28 (46.7)	28 (46.7)	
M:F	2.5:1.2	2.1:1.75	2:1.75	
**Serum p53 levels via ELISA(pg/mL)**				
Range	303–3898	392–4249	149–596	<0.001^a^
Mean ± SD	1022 ± 496	1211 ± 796	340 ± 120	
**Categorical distribution of serum p53 Ab levels (pg/mL) via ELISA, n (%)**				
0–400	2 (3.30)	1 (1.66)	40 (66.7)	<0.001^b^
401–800	14 (23.3)	20 (33.3)	20 (33.3)	
801–1400	39 (65.0)	23 (38.3)	–	
>1400	5 (8.30)	16 (26.6)	–	
**Serum p53 Ab status via ELISA, n (%)**				
Seronegative	2 (43.3)	1 (1.60)	40 (66.6)	<0.001^b^
Seropositive	58 (96.6)	59 (98.3)	20 (33.3)	
**p53 tissue immunoreactivity via Immunohistochemistry**				
Immunonegative	16 (26.6)	9 (15.0)	59 (98.3)	<0.001^b^
Immunopositive	44 (73.3)	51 (85.0)	1 (1.70)	

∗a = ANOVA; b = Pearson's Chi square test.

Among patients diagnosed with OPMDs, the most common sites of OPMDs development included the cheek mucosa, vestibule of the mouth, and retromolar trigone (63.3%, n = 38). Clinically, the most commonly occurring entity was oral leukoplakia (43.4%; n = 26), and histopathologically, squamous cell hyperplasia was the most common (61.7, n = 37) epithelial precursor lesion (Table 2).

**Table 2:** Clinico-pathological features of OPMDs regarding p53 seropositivity status and p53 protein expression.

Clinico-pathological features of OPMD	p53 immunoreactivity		Serum p53 Ab status	
	Negative n (%)	Positive n (%)	Negative n (%)	Positive n (%)
**Site of OPMD lesions**				
***p*-value**	**0.822**		**0.945**	
Lip (External and inner aspects of lip, commissure of lip)	–	3 (5.00)	–	3 (5.00)
Tongue	4 (6.70)	9 (15.0)	–	13 (21.7)
Gum (Upper and lower gum)	1 (1.70)	2 (3.30)	–	3 (5.00)
Floor of mouth (Anterior and lateral floor of mouth)	–	1 (1.70)	–	1 (1.70)
Palate (Hard and soft palate, uvula)	1 (1.70)	1 (1.70)	–	2 (3.30)
Other and unspecified parts of mouth (Cheek mucosa, vestibule of mouth, retromolar area)	10 (16.7)	28 (46.7)	2 (3.30)	36 (60.0)
**Clinical diagnosis of OPMDs**				
***p*-value**	0.35		0.944	
Oral Leukoplakia	5 (8.30)	21 (35.0)	1 (1.70)	25 (41.7)
Speckled leukoplakia	4 (6.70)	6 (10.0)	–	10 (16.7)
Oral Proliferative verrucous leukoplakia	1 (1.70)	2 (3.30)	–	3 (5.00)
Oral Lichen planus	4 (6.70)	8 (13.3)	1 (1.70)	11 (18.3)
Oral Erythroplakia	2 (3.30)	1 (1.70)	–	3 (5.00)
Snuff dippers keratosis	–	3 (5.00)	–	3 (5.00)
Actinic cheilosis	–	3 (5.00)	–	3 (5.00)
**Histological description of epithelial precursor lesions**				
***p*-value**	0.369		0.257	
Squamous cell hyperplasia	8 (13.3)	29 (48.3)	2 (3.35)	35 (58.3)
Oral epithelial dysplasia	8 (13.3)	15 (25.0)	–	23 (38.3)
**Grades of Binary system of OED**				
***p*-value**	0.672		∗	
Low risk lesion	5 (38.4)	8 (61.5)	–	13 (56.5)
High risk lesion	3 (30.0)	7 (70.0)	–	10 (43.5)
**Description of subepithelial Inflammatory infiltrate**				
***p*-value**	0.098		0.833	
Chronic inflammatory infiltrate	11 (18.3)	40 (66.7)	2 (3.30)	49 (81.7)
Acute on chronic inflammatory infiltrate	2 (3.30)	2 (3.30)	–	4 (6.70)
None	3 (5.00)	2 (3.30)	–	5 (8.30)

Among the 42 cases of OPMDs with p53 protein expression, 42 expressed both p53 antibodies and p53 protein accumulation in precancerous cells. Statistically significant differences were noted between p53 immunoreactivity in tissue and p53 antibody status among healthy individuals and cases (OPMDs and OSCC) [Table 3].

**Table 3:** p53 immunoreactivity and serum p53 autoantibodies status among healthy individuals and cases (OPMDs & OSCC).

Study Groups	Serum p53 Ab status	p53 immunoreactivity		Total n (%)	*p*-value∗
		Negative n (%)	Positive n (%)		
**p53 immunoreactivity and serum p53 autoantibodies status among OPMD & healthy individuals**					
Healthy individuals	Negative	40 (66.7)	–	40 (66.7)	<0.001
	Positive	19 (31.7)	1 (1.70)	20 (33.3)	
OPMDs	Negative	–	2 (3.30)	2 (3.30)	
	Positive	16 (26.7)	42 (70.0)	58 (96.7)	
**p53 immunoreactivity and serum p53 autoantibodies status among OSCC & healthy individuals**					
Healthy individuals	Negative	40 (66.7)	–	40 (66.7)	<0.001
	Positive	19 (31.7)	1 (1.70)	20 (33.3)	
OSCC	Negative	–	1 (1.70)	1 (1.70)	
	Positive	9 (15.0)	50 (83.0)	59 (98.3)	
**p53 immunoreactivity and serum p53 autoantibodies status among OPMD & OSCC**					
OPMDs	Negative	–	2 (3.30)	2 (3.30)	0.386
	Positive	16 (26.7)	42 (70.0)	58 (96.7)	
OSCC	Negative	–	1 (1.70)	1 (1.70)	
	Positive	9 (15.0%)	50 (83.3)	59 (98.3)	

∗Pearson's Chi-square test.

For OPMDs, the recorded predictive value for serum p53 antibodies was characterised by high sensitivity (96.6%) and low specificity (66.0%), while for tissue p53 immunoreactivity, it was 73.0% and 98.3%, respectively, with an accuracy of more than 80.0% (Table 4).

**Table 4:** Predictive value of tissue p53 (immunoreactivity and seroreactivity) of OPMDs regarding OSCC and healthy individuals.

Statistics	p53 immunoreactivity via immunohistochemistry		p53 Ab status via ELISA
	OPMD	OSCC	OPMD
Sensitivity	73.3%	85.0%	96.7%
	**95% CI (60.3**–**83.9)**	**95% CI (73.4**–**92.9)**	**95% CI (88.5**–**99.6)**
Specificity	98.3%	98.0%	66.7%
	**95% CI (91.1**–**100)**	**95% CI (91.1**–**100)**	**95% CI (53.3**–**78.3)**
Positive predictive value (PPV)	97.8%	98.0%	74.0%
Negative predictive value (NPV)	78.7%	86.0%	95.0%
Likelihood ratio of positive result (LR+)	44.0	51.0	2.82
Likelihood ratio of negative result (LR-)	0.27	0.15	0.06
Accuracy	85.0%	91.0%	81.0%
Misclassification rate	0.14	0.08	0.18
Diagnostic odd's ratio	162; **p < 0.001;** **95% CI (20.7**–**1270)**	334; **p < 0.001;** **95% CI (41.0**–**2729)**	46; p < 0.001; **95% CI (12.8**–**262)**

## Discussion

Among oral cancers, OSCC is reported as the most frequently occurring histopathological entity. OPMDs are disorders that usually develop into an oral epithelial malignancy if they go unnoticed.^1^^,^^4^ Finding biomarkers that can aid in predicting the malignant potential of OPMD for timely diagnosis of OSCC in early phases of carcinogenesis to improve the prognosis. In the present study, tissue p53 immunoreactivity and serum p53 antibody status were evaluated among cases of OPMDs and were compared with the findings in OSCC and healthy individuals to examine its role in the timely indication and conversion into oral malignancy.

Our study observed that most OPMDs cases included patients older than 60 years of age. This finding supports the observations reported by Mello et al., who conducted a systematic review and meta-analysis on the prevalence of OPMDs.^3^ Previous research has also indicated age as a prognostic indicator for OPMDs.^5^

In the present study, the observed male to female ratio among cases of OPMDs is in line with other studies conducted in Brazil (2.4:1.8),^3^ Australia (2.3:1.6),^28^ and India (2.5:1.5),^29^ and contrary to the studies by Mishra et al., which found an m:f ratio of 4.1:1.5,^30^ and Jagtap, who reported an almost equal ratio (1.9:1).^31^ Among females, research studies have observed a low occurrence of OPMDs but with a high rate of malignant transformation.^5^

In the present study, the recorded male sex and old age preponderance were in agreement with previous studies.^28^^,^^30^

Similar to the cases of OPMDs, the findings of the present study related to OSCC regarding age and gender are in agreement with the findings of other researchers.^1^^,^^23^^,^^26^

### p53 immunoreactivity

The present study found significantly high tissue p53 immunoreactivity among patients with OSCC, followed by OPMDs. Our research observed the same high frequency (73.3%) for tissue p53 immunoreactivity among cases of OPMDs, as reported by national and international studies (up to 80%).^23^^,^^32^^,^^33^

The finding of p53 immunostaining in suprabasal layers of only one tissue sample of normal oral mucosa is contrary to numerous studies that observed a complete absence of p53 protein expression, exclusively in all oral epithelial layers or present in the basal layer only.^33^, ^34^, ^35^, ^36^ However, a study by Cruz et al. observed p53 immunoreactivity in suprabasal layers of non-malignant mucosa, adjacent to the OSCC lesions.^37^ However, in the present study, only one subject expressed p53 in the oral mucosa, and he was categorised as male, above 50 years of age, and in the group of tobacco consuming individuals. Detection of p53 protein in normal oral mucosa is mostly absent due to the brief half-life of the wild type of p53 protein or due to expression of minimal quantity, which is difficult to detect on immunohistochemistry and which, if present, is mostly confined to the basal layer of epithelium.^38^ Furthermore, some researchers have indicated the detection of p53 protein in normal cells without malignant potential as a physiological response of cells to genotoxic stress.^27^

### p53 antibodies

Our study observed significantly raised the mean serum p53 antibody levels among patients with OSCC, followed by OPMDs and healthy individuals. The present study observed a high proportion of p53 antibodies seropositivity (96.6%) among cases of OPMDs (Table 1), which contrasted the frequency of detection of serum antibodies against p53 (0–30%) noted by Ralhan et al., Sainger et al. (6.60%), and Porrini et al. (0.00%).^11^^,^^39^^,^^40^ The present study reported a considerable number of healthy individuals (33.0%) expressing serum p53 antibodies (Table 1). Previous research has reported variable results regarding the seropositivity of p53 antibodies among healthy controls, ranging from 0.00% to 24.2%.^11^^,^^40^^,^^41^ This difference in the results is most probably due to the demographic variation of the population under study.

The presence of serum p53 antibodies among healthy individuals (n = 20) in our study is noteworthy. The possible explanation for p53 antibody seropositivity among healthy individuals may be consistent with findings observed in high-risk individuals, explained with the presence of p53 antibodies several years before the clinical detection of malignancy.^17^^,^^42^

Furthermore, the physiological response of cells to genotoxic stress may be due to a defect in the degradation pathway leading to the accumulation of non-functional p53 phosphoprotein.^19^^,^^25^^,^^27^^,^^37^^,^^43^

### Tissue p53 expression and serum p53 antibodies among healthy individuals and cases

A highly significant difference (*p* = 0.001) in p53 tissue immunoreactivity was observed between healthy individuals and OPMD cases (Table 1). These findings have also been supported by Basheer et al. (*p* = 0.003)^35^ and Hadzi-Mihailovic et al. (0.018),^32^ and contrasting observations by Purwaningsih et al. (*p* = 0.091).^33^

In our study, statistically significant differences in serum p53 antibody status were observed among healthy individuals and patients with OPMD. These findings are in line with the observations noted by Ralhan et al.^43^ but contrast the observations of Porrini et al. (possibly due to the recruitment of most of the cases of oral lichen planus) and Sainger et al. (possibly due to the recruitment of most cases of oral submucosal fibrosis).^11^^,^^40^ The present study found a statistically significant relationship between serum p53 antibody status and tissue p53 immunoreactivity among healthy controls and patients with OPMDs.

### Predictive value of tissue p53 immunoreactivity

Our study observed sensitivity of 73.3%, showing that the test correctly recognised 44(n) out of 60(n) cases of OPMD and did not recognise 26.7% of them. The recorded specificity (98.3%) by the same test indicates that it has the ability to effectively detect disease-free subjects, and only 1.70% of false positives were not detected. These findings are in contrast to Purwaningsih et al. (possible reasons may include the small sample size and lack of sub-classification of cases with OED and without dysplasia) and Basheer et al. (possible reasons may include the small sample size and the fact that among numerous OPMDs, only cases of oral lichen planus were recruited) who reported low sensitivity of 55.0% and 25%, respectively,^33^^,^^35^ and high specificity (100%) and Hadzi-Mihailovic et al.^32^ who reported a sensitivity of 80.0% and specificity of 53%. In the present study, a PPV of 97.8% showed that 2.22% of the results were false positive, while 78.7% of NPV indicated that a negative test dismissed the disease. The LR+ of 44 indicated that the test could provide a strong clue to confirm a diagnosis. However, the LR- of 0.27 revealed that the test provided weak evidence to reject a diagnosis. A high diagnostic odds ratio (DOR) revealed that the test had a better discrimination test performance. A misclassification rate of 14% indicated the proportion of subjects who were incorrectly categorised by the test.^44^^,^^45^

Among cases of OSCC, the test was able to identify 85% of true diseased subjects while 2% of false-positive subjects were not recognised effectively.

### Predictive value of p53 antibodies seroreactivity

The present study noted that the test had a high sensitivity (96.0%) and a low specificity value (66.0%), which indicates that it was not able to detect disease-free subjects effectively. These observations support the findings of Ralhan et al., Sanger et al., and Porrini et al., who observed low sensitivity and high specificity.^11^^,^^40^^,^^43^ Moreover, PPV revealed that 26.0% of the results were false positive. An NPV of 95.0% disclosed that those subjects who tested negative had a 95% possibility of not having the disease. LR+ of 2.82 indicated that the test provided weak evidence to confirm a diagnosis while the LR- (0.06) provided strong evidence to reject a diagnosis, as it was less than 0.1. A high DOR showed that the test had a better discrimination test performance. The misclassification rate (18.0%) showed the proportion of subjects who were incorrectly categorised by the test.^44^^,^^45^

The sensitivity and specificity of serum p53 antibodies in the same cases (OSCC) were 98.0% (95% CI [91.06–99.96]) and 66% (95% CI [53.31–78.31]), respectively, as reported in our previous study.^22^

The development of a simple, rapid, and non-invasive method for predicting the prognosis of potentially malignant precursors in the early stages is the key to improving the prognosis of OSCC.

OPMD cases with tissue p53 immunopositivity and p53 seropositivity should be monitored more closely than p53 negative cases. These tests can possibly act as potential post-operative monitoring markers for predicting the prognosis or malignant potential of OPMDs and hence improve the prognosis of OSCC by facilitating its early phase diagnosis in high-risk individuals. Furthermore, these tests can serve as useful markers for routine screening of asymptomatic high-risk patients. Additionally, it is recommended that next to the assessment of serum, saliva can also be evaluated for the detection of p53 autoantibodies, and the results of both can be correlated with p53 protein expression among cases of OPMDs. Further studies should evaluate the prognostic value of serum p53 autoantibodies in OPMD cases with long-term follow-up.

## Conclusion

This study found that serum p53 antibody status could be used as a screening tool for predicting the OPMDs prognosis because of its high sensitivity and non-invasiveness. Furthermore, for high-risk OPMD lesions, p53 immunoreactivity may be recommended for predicting the probable malignant potential of OPMDs, which will therefore improve the prognosis of OSCC by intercepting the disease in the preclinical cancerous stage.

## Source of funding

This research did not receive any specific grant from funding agencies in the public, commercial, or not-for-profit sectors.

## Conflict of interest

The authors have no conflicts of interest to declare.

## Ethical approval

Ethical permission to conduct this study was obtained from the institutional review board of the first author's institute (Institutional Review Board of Prime Foundation Pakistan [IRB approval no. PMC/IRB/2015-003]; Dated: 26th August 2015). Written consent from all the prticipants of the study was obtained.

## Authors contributions

The chief investigator of this project ASK attests that this study was conducted by the authors whose names were mentioned. The specific contributions are as follows: ASK was involved in the conceptual design of the work, data collection, data management, and manuscript writing under the chief supervision of SA and co-supervision of Z. NS analysed and statically interpreted the data. MH provided key assistance in laboratory work, and AKS assisted in the histological review. All authors have critically reviewed and approved the final draft and are responsible for the content and similarity index of the manuscript.
